# Plasmon-Enhanced Multiphoton Polymer Crosslinking for Selective Modification of Plasmonic Hotspots

**DOI:** 10.1021/acs.jpcc.4c05936

**Published:** 2024-10-22

**Authors:** Yevhenii M. Morozov, Nestor Gisbert Quilis, Stefan Fossati, Laura De Laporte, Claudia Gusenbauer, Andreas Weber, Jose Luis Toca-Herrera, Fiona Wiesner, Ulrich Jonas, Jakub Dostalek

**Affiliations:** † Center for Health & Bioresources, AIT-Austrian Institute of Technology, Giefinggasse 4, 1210 Vienna, Austria; ‡ Biosensor Technologies, AIT-Austrian Institute of Technology, Konrad-Lorenz-Strasse 24, 3430 Tulln an der Donau, Austria; § FZU-Institute of Physics, Czech Academy of Sciences, Na Slovance 2, 182 21 Prague, Czech Republic; ∥ DWI-Leibniz Institute for Interactive Materials, Forckenbeckstrasse 50, D-52074 Aachen, Germany; ⊥ Institute for Technical and Macromolecular Chemistry, RWTH Aachen University, Worringerweg 1-2, D-52074 Aachen, Germany; # Institute of Applied Medical Engineering, Department of Advanced Materials for Biomedicine, RWTH Aachen University, Forckenbeckstraße 55, D-52074 Aachen, Germany; ∇ Institute of Wood Technology and Renewable Materials, University of Natural Resources and Life Sciences, Vienna, Konrad-Lorenz-Strasse 24, 3430 Tulln an der Donau, Austria; ○ Institute of Biophysics, University of Natural Resources and Life Sciences, Vienna, Muthgasse 11/II, 1190 Vienna, Austria; ◆ Macromolecular Chemistry, Department of Chemistry and Biology, University of Siegen, Adolf Reichwein-Straße 2, 57074 Siegen, Germany; ¶ LiST-Life Sciences Technology, Danube Private University, Viktor-Kaplan-Strasse 2, 2700 Wiener Neustadt, Austria

## Abstract

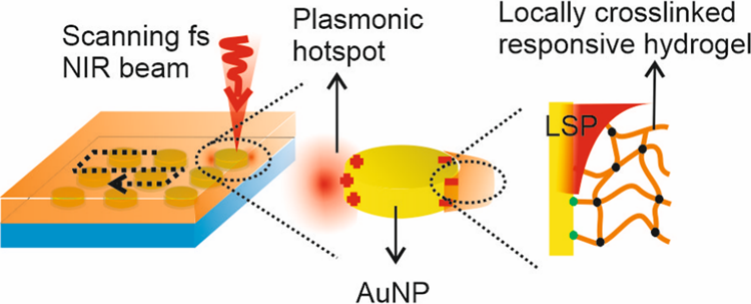

A novel approach to selectively modify narrow subareas of metallic nanostructures adjacent to plasmonic hotspots, where strong electromagnetic field amplification occurs upon localized surface plasmon (LSP) excitation, is reported. In contrast to surface plasmon-triggered polymerization, it relies on plasmonically enhanced multiphoton crosslinking (MPC) of polymer chains carrying photoactive moieties. When they are contacted with metallic nanostructures and irradiated with a femtosecond near-infrared beam resonantly coupled with LSPs, the enhanced field intensity locally exceeds the threshold and initiates MPC only at plasmonic hotspots. This concept is demonstrated by using gold nanoparticle arrays coated with two specifically designed polymers. Local MPC of a poly(*N*,*N*-dimethylacrylamide)-based copolymer with an anthraquinone crosslinker is shown via atomic force microscopy. Additionally, MPC is tested with a thermoresponsive poly(*N*-isopropylacrylamide)-based terpolymer. The reversible thermally induced collapse and swelling of the MPC-formed hydrogel at specific nanoparticle locations are confirmed by polarization-resolved localized surface plasmon resonance (LSPR) spectroscopy. These hybrid metallic/hydrogel materials can be further postmodified, offering attractive characteristics for future spectroscopic/bioanalytical applications.

## Introduction

Optical nanoantennas that allow for nanoscale confinement of the electromagnetic field play a pivotal role in numerous important research fields and application domains relying on optical spectroscopy and photochemical reactions in tightly confined volumes.^1−5^ For example, such nanoantennas have enabled the study of fundamental optical phenomena at the single emitter level (such as organic fluorophore molecules, quantum dots, or nitrogen-vacancy centers in diamonds),^6−9^ in single biomolecule interaction analysis by using labeling with such fluorescent species,^10^ or on hot electron-facilitated catalysis.^11,12^ Metallic nanostructures and nanoparticles (NPs) represent key building blocks for the construction of optical nanoantennas. The resonant excitation of localized plasmons (LSPs) on such metallic NPs allows for deep subwavelength confinement of light energy due to collective oscillations of electron density and the associated electromagnetic field.^13,14^ The optical excitation of LSPs is accompanied by a strong enhancement of the incident light intensity, which is not homogeneously distributed around metallic NPs but occurs at specific spatially distinct regions (e.g., edges/tips or gaps between closely arranged metallic objects) that are commonly referred to as “plasmonic hotspots”.^15^ Therefore, various strategies to spatially control the attachment of functional chemical moieties at these regions have been pursued for precise control and to maximize the efficiency of their interaction with LSPs.^15−18^ These include the use of photoresist mask windows made by electron beam lithography to overlay with plasmonic hotspots,^19^ development of orthogonal surface chemistries,^16,20^ and employment of a static electric field gradient for attracting the species at sharp parts of metallic nanostructures.^21^ The use of chemical reactions that are locally triggered by LSPs has been explored based on the LSP-generated hot electrons,^22^ two-photon polymerization,^23^ and by using multiphoton absorption-activated linkers for docking of proteins^24−26^ and local photocrosslinking of protein hydrogel.^27^ In addition, LSP-induced polymerization has been shown to allow for local attachment of nanoscale photoresist features overlapping with plasmonic hotspots^28−32^ and later it was intensively used for the preparation of hybrid polymer/metal materials and probing of local chemical reactions.^33−37^

Multiphoton absorption photochemistry has become particularly important in mask-less lithography^38,39^ developed for the preparation of complex structures with a spatial resolution down to a hundred nanometers.^40^ Up to now, multiphoton lithography techniques have been utilized for the fabrication of objects with a well-controlled three-dimensional geometry serving in diverse areas including micro-optics,^41−43^ microrobotics,^44,45^ tissue engineering,^46−50^ and drug delivery^51^ to mention a few examples. The vast majority of materials that are structured with the help of multiphoton absorption lithography rely on a polymerization process.^52^ Typically, a femtosecond (fs) pulsed laser beam with a near-infrared (NIR) wavelength is focused and scanned through a liquid monomer to initiate polymerization in a small focal volume, where the threshold for initiation is exceeded.^53^ Only along the path of the NIR beam are the included photoinitiator molecules excited by the sequential absorption of photons to initiate a free-radical polymerization reaction locally solidifying the liquid photoresist. Besides multiphoton lithography relying on a polymerization mechanism, a recently reported alternative is based on multiphoton crosslinking (MPC) of soluble polymers employed as solid thin films.^54^ Their polymer backbones contain photoactive moieties that were excited by a scanned focused NIR beam to induce MPC, resulting in thermoresponsive biofunctional hydrogels when a postmodification step is utilized.^55^ The MPC process was investigated in dependence on the type of crosslinker and its means of conjugation to the polymer backbone.^55^ This crosslinking-based approach, in contrast to the more frequently used polymerization, allows for the creation of structures from a compact dry polymer layer, eliminating the risk of residual monomer and/or photoinitiator leakage during swelling and offering the advantage of higher chemical precision when incorporating multiple functionalities during the polymer presynthesis stage.

In the present work, we report on a new generic approach to chemically modify narrow zones of metallic nanostructures, where plasmonic hotspots occur. The strategy employs polymer chains with incorporated photoactive crosslinkers that can be triggered by the multiphoton absorption process for their simultaneous attachment and crosslinking at the plasmonic hotspot where the MPC threshold is locally reached. To the best of our knowledge, in contrast to the plasmonically driven polymerization, this type of plasmonically mediated photochemistry—plasmon-enhanced multiphoton crosslinking (PE-MPC)—has not yet been reported, despite its potential for preparing well-controlled hybrid metal/hydrogel nanomaterials. This method leverages the already established multiphoton lithography tools and offers the capability for selective local postmodification of the resulting structures. The reliability of the approach is demonstrated by using two types of polymers that form locally attached hydrogel nanostructures at plasmonic hotspots after swelling in aqueous media. The successful preparation of such materials is documented for optimized writing conditions by atomic force microscopy (AFM) measurements of the local topography and Young’s modulus and by characterizing the nanoscopic domains of the thermoresponsive hydrogel matrix with polarization-resolved localized surface plasmon resonance (LSPR) measurements.

## Materials and Methods

### Materials

Poly(DMAA-*co*-AAHAQ) was synthesized as previously reported.^54^ Poly(NIPAAm-*co*-MAA-*co*-BPQAAm) was synthesized as previously reported.^55^ Benzophenone disulfide (BPDiS) was synthesized as reported elsewhere.^56^ S1805 resist and AZ303 developer were purchased from Micro resist Technology GmbH (Germany). Dimethyl sulfoxide (DMSO) was purchased from Sigma-Aldrich, Austria.

### Preparation of Hydrogel-Coated Gold Particle Arrays

Periodic arrays of gold nanoparticles (AuNPs) with a diameter *D* = 185 ± 10 nm and period Λ = 400 nm (in the case of structures with poly[DMAA-*co*-AAHAQ]) and with a diameter *D* = 165 ± 10 nm and period Λ = 400 nm (in the case of structures with poly[NIPAAm-*co*-MAA-*co*-BPQAAm] terpolymer) were employed as plasmonic substrates. Briefly, a BK7 glass slide was coated with a 2 nm thick chromium layer and 50 nm thick gold layer by thermal evaporation (UNIVEX 450, Helmut Heller GmbH, Germany). Then, an S1805 positive photoresist (1:2 ratio diluted with propylene glycol monomethyl ether acetate) was spun on the gold surface, yielding a layer with a thickness of 120 nm. A periodic interference pattern was recorded into the photoresist layer by employing a He–Cd laser (λ = 325 nm) with Lloyd’s mirror setup as previously reported by our group.^57^ The well-defined resist particles with subwavelength dimensions were etched by using the AZ-303 developer (1:15 ratio with deionized water). Finally, the inscribed pattern was transferred to the gold film by using dry etching with an argon milling system (IonSys 500, Roth&Rau, Germany). The remaining photoresist was removed by applying an oxygen plasma treatment. The prepared plasmonic substrate was immersed overnight in a 1 mM solution made of BPdiS dissolved in DMSO in order to form a self-assembled monolayer on the pristine gold surface. The photocrosslinkable polymer thin film was subsequently spun (from ethanolic solution with 3 and 2% w/w of poly(DMAA-*co*-AAHAQ) and poly(NIPAAm-*co*-MAA-*co*-BPQAAm), respectively) on top of the AuNP arrays at a rate of 2000 rpm for 60 s and dried overnight in a vacuum oven at *T* = 50 °C.

### Plasmon-Enhanced Multiphoton Crosslinking of Poly(DMAA-*co*-AAHAQ)

Poly(DMAA-*co*-AAHAQ) was deposited on the top of AuNP arrays carried by a glass substrate, as described above. The substrate was mounted into the Photonic Professional (GT) system from Nanoscribe GmbH for the recording step (with a wavelength of the emitted light centered at 780 nm),^58^ with the surface carrying the plasmonic architectures facing a clean coverslip. The interface was selected by focusing on the surface with the gold nanoparticle arrays, and the correct *z* plane was set by recording square features with the highest writing speed and laser power *P*_L_ and visually observing the recording of the micrometer-size features with the equipped microscope camera. Once the proper interface was found, a recording script was loaded and squares (100 × 100 μm^2^) with different parameters were inscribed. The scan speed was varied between 10,000 and 2500 μm s^–1^ and the laser power *P*_L_ = 2–5 mW. After the recording step, the unbound polymer was removed by rinsing with ethanol.

### Plasmon-Enhanced Multiphoton Crosslinking of Poly(NIPAAm-*co*-MAA-*co*-BPQAAm)

Poly(NIPAAm-*co*-MAA-*co*-BPQAAm) was deposited on the top of AuNP arrays carried by a glass substrate, as described above. The substrate was mounted into a home-built general-purpose multiphoton absorption lithography setup for the recording step (details of the setup can be found in refs (55,59)). The wavelength of the emitted light of the home-built setup is centered at 785 nm. After the selection of the correct *z* plane, a rectangular area of 75 × 50 μm^2^ was recorded. The laser power *P*_L_ was varied between 10 and 15 mW, and the dwelling time *t*_d_ was varied between 1 and 5 s.

### Morphological Characterization

For topography measurements of the AuNP arrays with attached polymer networks, AFM height topography was acquired with the help of a Bruker’s Dimension Icon (Bruker) instrument operated in tapping mode. For all measurements, triangular silicon nitride SCANASYST-AIR cantilevers (Bruker) with a nominative spring constant *k* of 0.4 N/m, a resonance frequency of 70 kHz (in air), and a tip radius of 2 nm were used. Before each experiment, the true spring constant in air was determined via thermal tuning calibration after determining the deflection sensitivity. The AFM measurements in water were carried out with a Nanowizard (JPK III, Germany) instrument equipped with the inverse microscope and a liquid chamber. Imaging of the samples in water was performed in contact mode and quantitative imaging (QI) mode (0.3 N/m cantilevers with a 10 nm tip radius). The mapping to determine the mechanical properties was conducted in force volume mode both in water and in air (force curve at each pixel with 10 μm/s, maximum force of 5 nN, up to 200 × 200 resolution for a 2 × 2 μm image). Young’s modulus was determined by fitting the approach section with the elastic model (Hertz model with Sneddon extension for geometry). Cantilever properties: *k* = 9 N/m (±2), resonance frequency 150 kHz, beam shaped, 175 μm, Al coating on the backside, OPUS shape (tetrahedral), radius below 7 nm, 35° back angle.

### Electromagnetic Simulations

The Ansys Lumerical FDTD numerical simulation package was used for all electromagnetic modeling. Geometrical parameters for arrays of AuNPs were used according to those mentioned in the main text and the Supporting Information. Below the AuNPs, a chromium layer with a thickness of 2 nm was assumed. A dielectric medium with a refractive index of 1.5 (nondispersive) representing the BK7 glass was used as a substrate. As a superstrate, a medium with a refractive index of 1.45 (nondispersive) representing the polymer in a dry state was used. A plane wave made normally incident at the structure was used as an excitation source. Periodic (along the *x*- and *y*-axes) and perfectly matched layer absorbing (at the *z*-axis, 1.2 μm above and below the structure) boundary conditions were applied. Au and Cr optical properties were taken from the literature.^60,61^

## Results and Discussion

### Design of Polymers and Plasmonic Nanostructures

**Figure 1:**
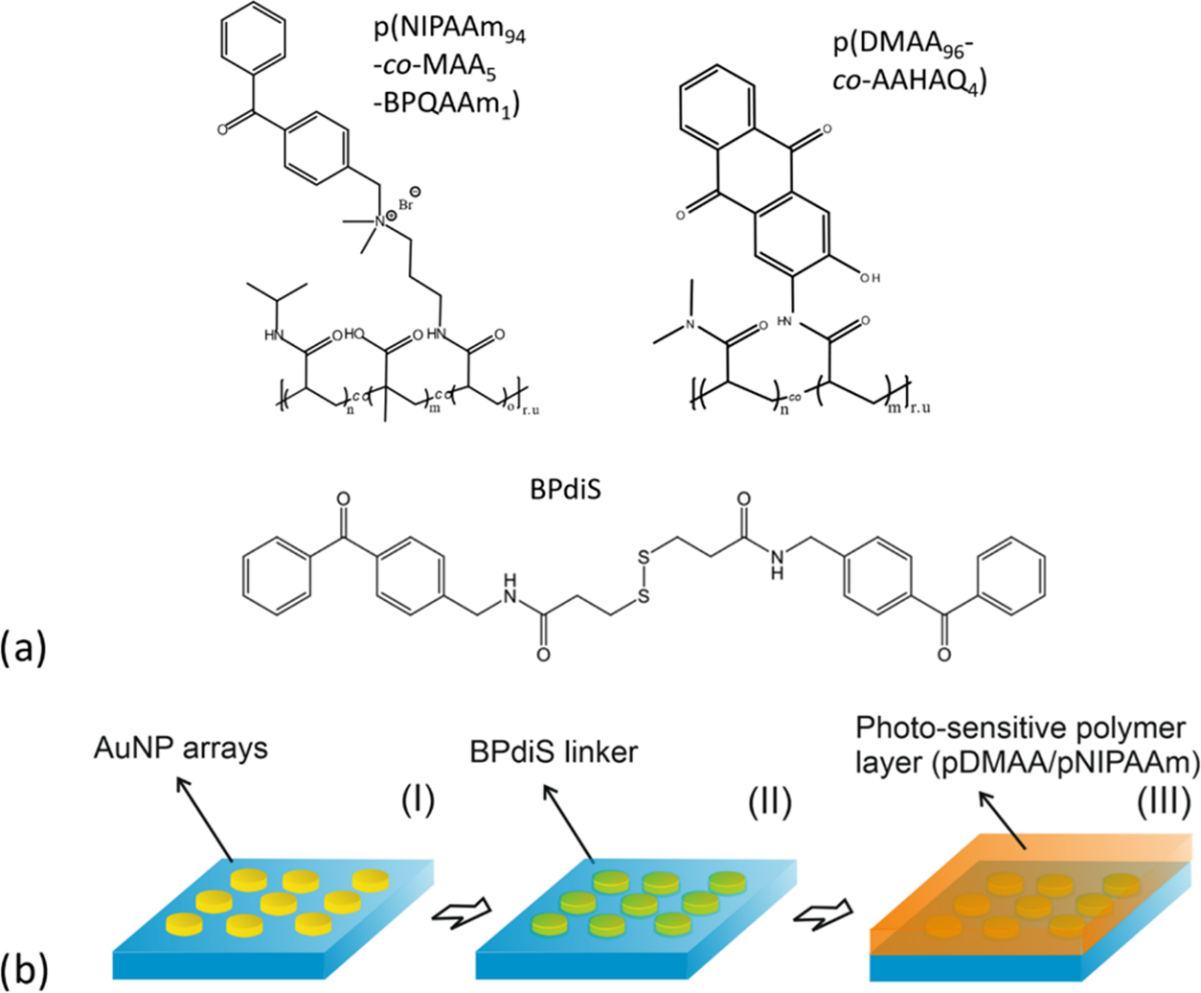
(a) Chemical structure of photocrosslinkable poly(DMAA_96_-*co*-AAHAQ_4_) and poly(NIPAAm_94_-*co*-MAA_5_-*co*-BPQAAm_1_) polymers and BPdiS linker and (b) schematics of the preparation procedure for the Au nanoparticle arrays coated with photocrosslinkable polymers.

The proposed concept was pursued with two types of photocrosslinkable polymers shown in Figure 1a, which form a polymer network via MPC and subsequently a hydrogel after swelling with water. Details on the mechanism of the photoinitiated crosslinking mechanism was the subject of previous studies,^54,55^ and the copolymer poly(*N*,*N*-dimethylacrylamide-*co*-acylamido-3-hydroxyanthraquinone), abbreviated as poly(DMAA_96_-*co*-AAHAQ_4_), was chosen for the preparation of rigid hydrogel structures. It is ascribed to an apparently higher crosslink density due to a high ratio of anthraquinone-based photocrosslinking units (4% AAHAQ polymerization feed) in the polymer backbone. In addition, the terpolymer poly(NIPAAm_94_-*co*-MAA_5_-*co*-BPQAAm_1_) was prepared by copolymerization of *N*-isopropylacrylamide (NIPAAm) with [(4-benzoylphenyl)methyl]dimethyl[3-(prop-2-enamido)propyl]azanium bromide (BPQAAm) and methacrylic acid (MAA). This polymer carried low amounts of photocrosslinking units (1% BPQAAm in the polymerization feed) and allowed for the preparation of softer thermoresponsive hydrogel features. It was possible to modulate the degree of swelling for this pNIPAAm-based hydrogel with lower critical solution temperature (LCST) behavior by changing the temperature from below the volume phase transition temperature (VPTT), where the polymer network contacting water is in a swollen state, to above the VPTT, where it collapses. In further experiments, the polymers were crosslinked by using a focused beam of pulsed femtosecond (fs) lasers emitting at a wavelength of λ_L_ = 780–785 nm that was scanned over the substrate with a home-built setup^55,59^ and alternatively by a commercially available Photonic Professional (GT) system (Nanoscribe GmbH, Germany).^58^

The overall preparation route of the substrates used in PE-MPC experiments is summarized in Figure 1b. Briefly, arrays of cylindrically shaped AuNPs were prepared by using UV laser interference lithography in combination with Ar ion milling (step I).^57^ Afterward, the gold surface of AuNPs was reacted with 3,3′-disulfanediylbis(*N*-(4-benzoylbenzyl)propanamide) (BPdiS) to serve as a photoactive linker (step II). Finally, a photocrosslinkable polymer layer (pDMAA- or pNIPAAm-based) with a thickness *t* of about 100 nm was deposited over the AuNP arrays (step III) prior to MPC with a focused femtosecond NIR laser beam.

**Figure 2:**
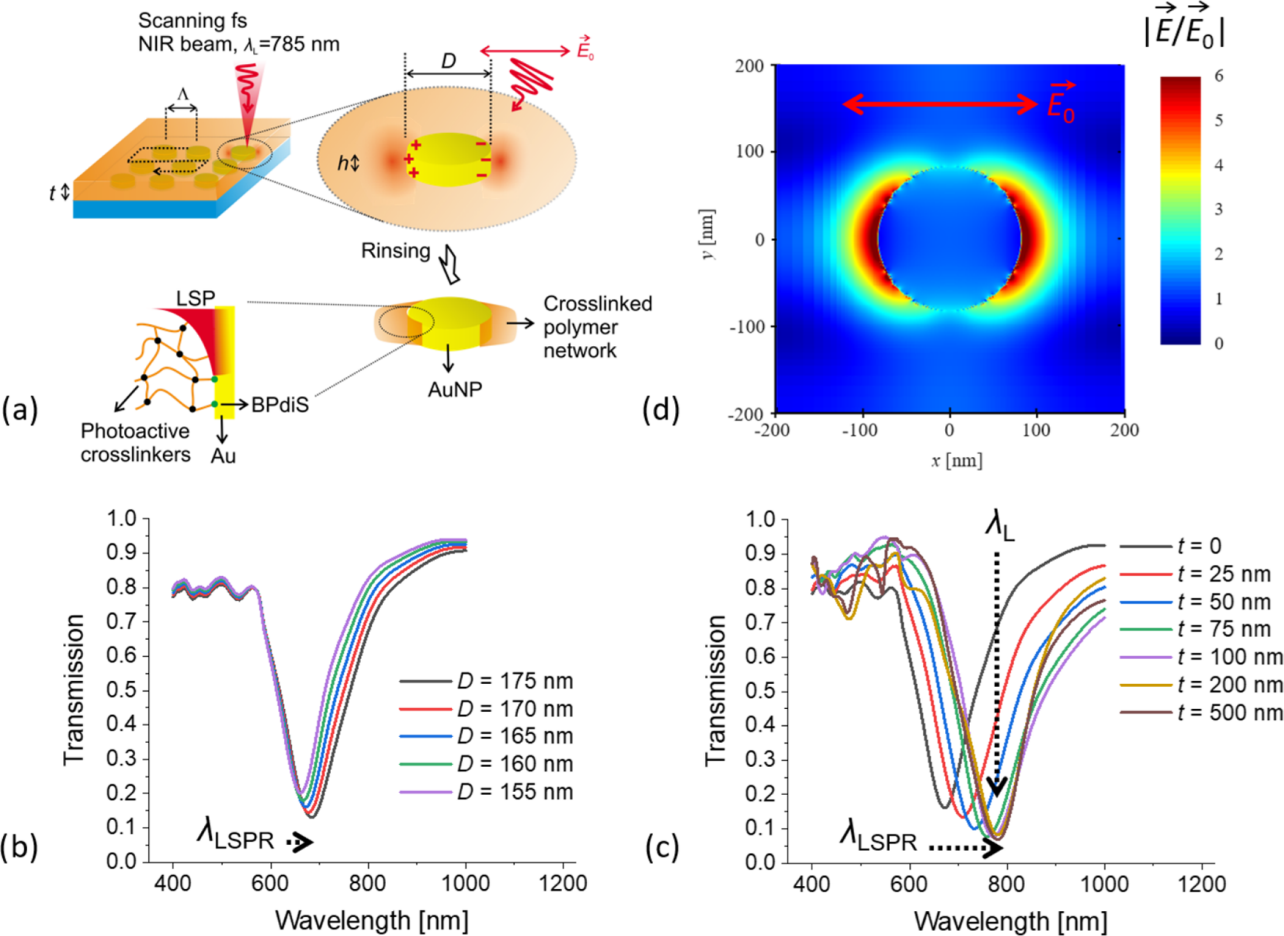
(a) Schematics of the crosslinking of polymer layer via PE-MPC. (b) Simulations of transmission spectra for AuNP arrays with varied Au disk diameters *D* = 155–175 nm and when the polymer overlayer is absent (thickness *t* = 0). (c) Simulated transmission spectra for *D* = 165 nm when the polymer overlayer thickness *t* is increased up to 500 nm. (d) Simulated near-field distribution of the electric field intensity amplitude upon resonant excitation of LSPs on AuNP with *D* = 165 nm at λ_L_ = 785 nm and *t* = 100 nm. In all simulations, the period of the AuNP arrays was Λ = 400 nm.

The MPC process of the polymer chains at plasmonic hotspots was induced by scanning a focused NIR femtosecond laser beam of a wavelength λ_L_ over the polymer-embedded AuNP arrays, as schematically presented in Figure 2a. The AuNPs are made resonant at a wavelength of λ_LSPR_ that is coincident with that of the NIR fs laser beam λ_L_. For the optimum scanning speed and intensity of the NIR fs laser beam *P*_L_, the resonant excitation of LSPs increases the electromagnetic field intensity above the threshold of the (nonlinear) multiphoton absorption process that activates crosslinker moieties (BPQAAm or AAHAQ) and allow for the formation of the surface-attached polymer network. The laser beam intensity *P*_L_ is chosen such that the NIR electromagnetic field intensity further away from the metallic nanostructure surface stays below the threshold. Therefore, any polymer chains outside the plasmonic hotspots are not crosslinked and upon rinsing with a solvent are washed away. This procedure and parameter optimization are described in our previous work,^55^ and it is also important to note that it is crucial to not exceed the threshold, above which the structure is damaged by increased temperature (see Figure S1, where certain laser power and writing speed values resulted in the structural burning of the poly[DMAA_96_-*co*-AAHAQ_4_] polymer).

The geometry, which enables matching of the LSPR wavelength λ_LSPR_ with that of NIR fs laser λ_L_, was identified by using finite-difference time-domain (Ansys Lumerical FDTD) simulations. The optimum Au disk diameter *D* and thickness of the polymer overlayer *t* were determined for cylindrically shaped AuNPs with a height of *h* = 50 nm when arranged in arrays with a period of Λ = 400 nm. As can be seen in Figure 2b, the resonant excitation of LSPs manifests itself as a narrow absorption band in the transmission spectrum with its minimum corresponding to λ_LSPR_. The resonant wavelength λ_LSPR_ shifts from 650 to 690 nm when increasing the AuNP diameter *D* from 155 to 175 nm in a structure without the polymer layer (thickness *t* = 0). Figure 2c shows that the presence of the polymer overlayer red-shifts the resonant wavelength λ_LSPR_ and for the AuNP diameter *D* = 165 and *t* > 100 nm, it yields λ_LSPR_ = 780–790 nm close to the laser wavelength λ_L_. An additional set of FDTD simulations was run to simulate the near-field distribution of electric field intensity upon the resonant excitation of LSPs at λ_L_ = λ_LSPR_ = 785 nm for this geometry. The cross section presented in Figure 2d shows that the peak enhancement of the electric field amplitude |*E*| reaches ∼6 with respect to the incident field |*E*_0_|. It exhibits a two-lobe distribution profile that is characteristic of a dipole LSP mode aligned in the direction of the incident field polarization. (A complete set of numerical results for the AuNP array with varying parameters can be found in Figure S2.)

### Topography and Young’s Modulus Measurements

**Figure 3:**
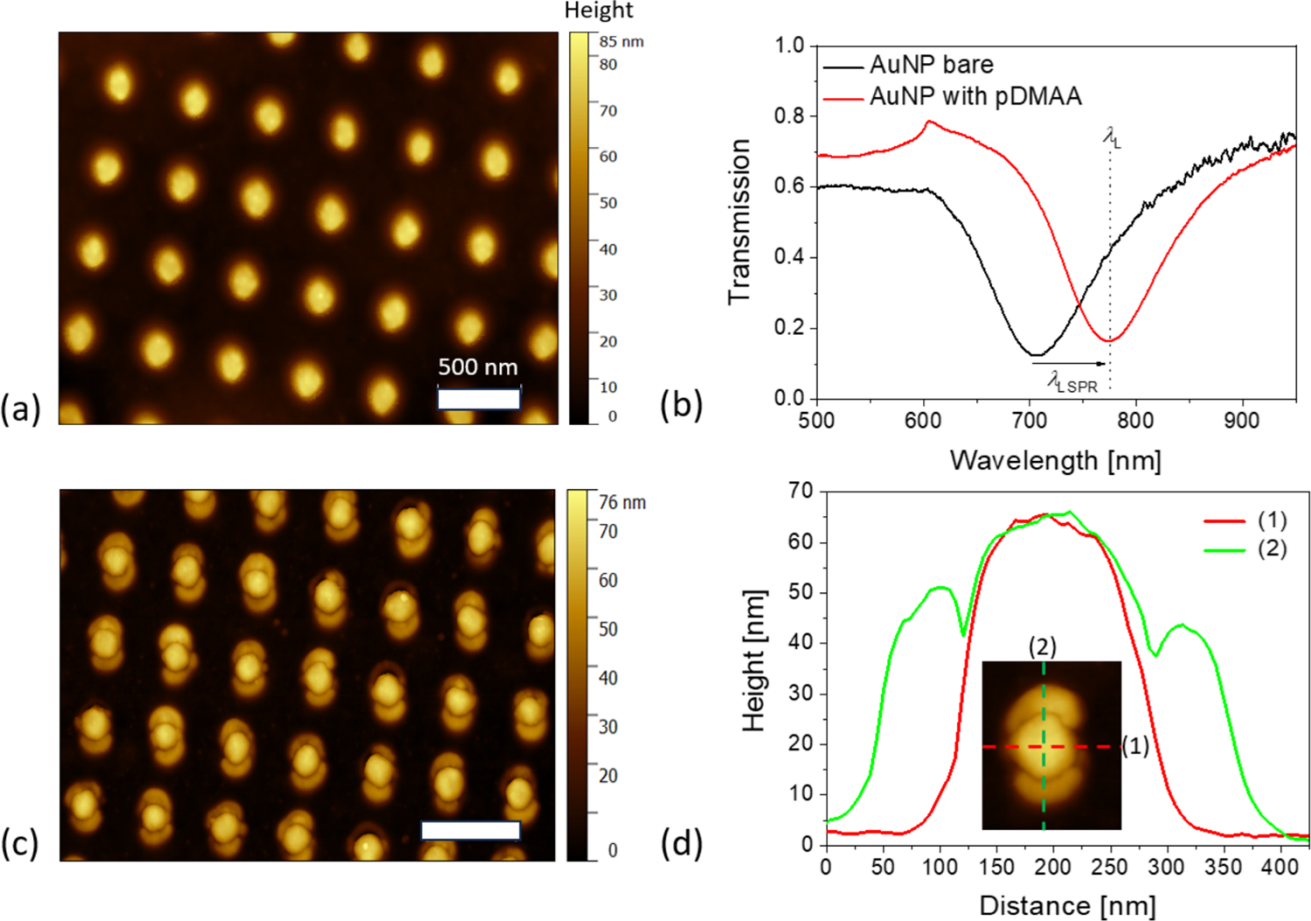
(a) AFM topography of the bare AuNP arrays and (b) measured LSPR transmission spectra before (black line) and after the coating (red line) with an uncrosslinked poly(DMAA_96_-*co*-AAHAQ_4_) layer of thickness *t* = 154 nm. (c) AFM height topography image of the same AuNP arrays after PE-MPC of poly(DMAA_96_-*co*-AAHAQ_4_) and rinsing. (d) Respective cross sections for individual AuNPs. All AFM measurements were performed in the air.

AFM was first used for the observation of morphology and elasticity of the poly(DMAA_96_-*co*-AAHAQ_4_) polymer networks attached to AuNP plasmonic hotspots. In this experiment, AuNP arrays with a diameter *D* of 185 ± 10 nm, a height of *h* = 50 nm, and a period of Λ = 400 nm were used with the respective height topography shown in Figure 3a. After depositing the poly(DMAA_96_-*co*-AAHAQ_4_) copolymer layer with a thickness of *t* = 154 nm (determined with the surface plasmon resonance measurement; see Figure S3), the LSPR wavelength of the AuNP array was shifted from λ_LSPR_ = 700 to 780 nm as confirmed by optical transmission spectroscopy (see Figure 3b). This λ_LSPR_ value is close to λ_L_ of the NIR beam of the used multiphoton lithographer, and it agrees with the previous simulations. AuNP arrays coated with poly(DMAA_96_-*co*-AAHAQ_4_) overlayer were scanned “line-by-line” with a focused NIR fs laser beam using a power of *P*_L_ = 4 mW and a writing speed of 2.5 × 10^3^ lines per second (by Nanoscribe Photonic Professional GT system). This condition was below the threshold for MPC without plasmonic amplification (Figure S1). After the MPC recording and ethanol rinsing step, the patterned structures were characterized by AFM, and Figure 3c shows the obtained height topography with detailed cross sections presented in Figure 3d. The successful local attachment of the polymer network changed the shape of the individual AuNPs, which became elongated along one axis, forming two apparent lobes stretching to opposite directions into a distance of 50 nm from the AuNP wall (defined as the distance where the height drops to half of its maximum; see Figure 3d). Such a shape can be attributed to the area of the plasmonic hotspot as simulated by FDTD and presented in Figure 2d. For the same AuNP, these simulations predict that the enhanced field intensity reaches a distance of about 50 nm (from the Au wall where the electric field amplitude drops to half of its magnitude; see Figure S2a) that is close to the experimental results. The orientation of the lobes is expected to be aligned with the polarization of the incident NIR fs beam, and irregularities in the shape can be attributed to variations in the plasmonic properties of individual AuNP associated with their polycrystalline nature^62^ and local deviations in shape.^57^

**Figure 4:**
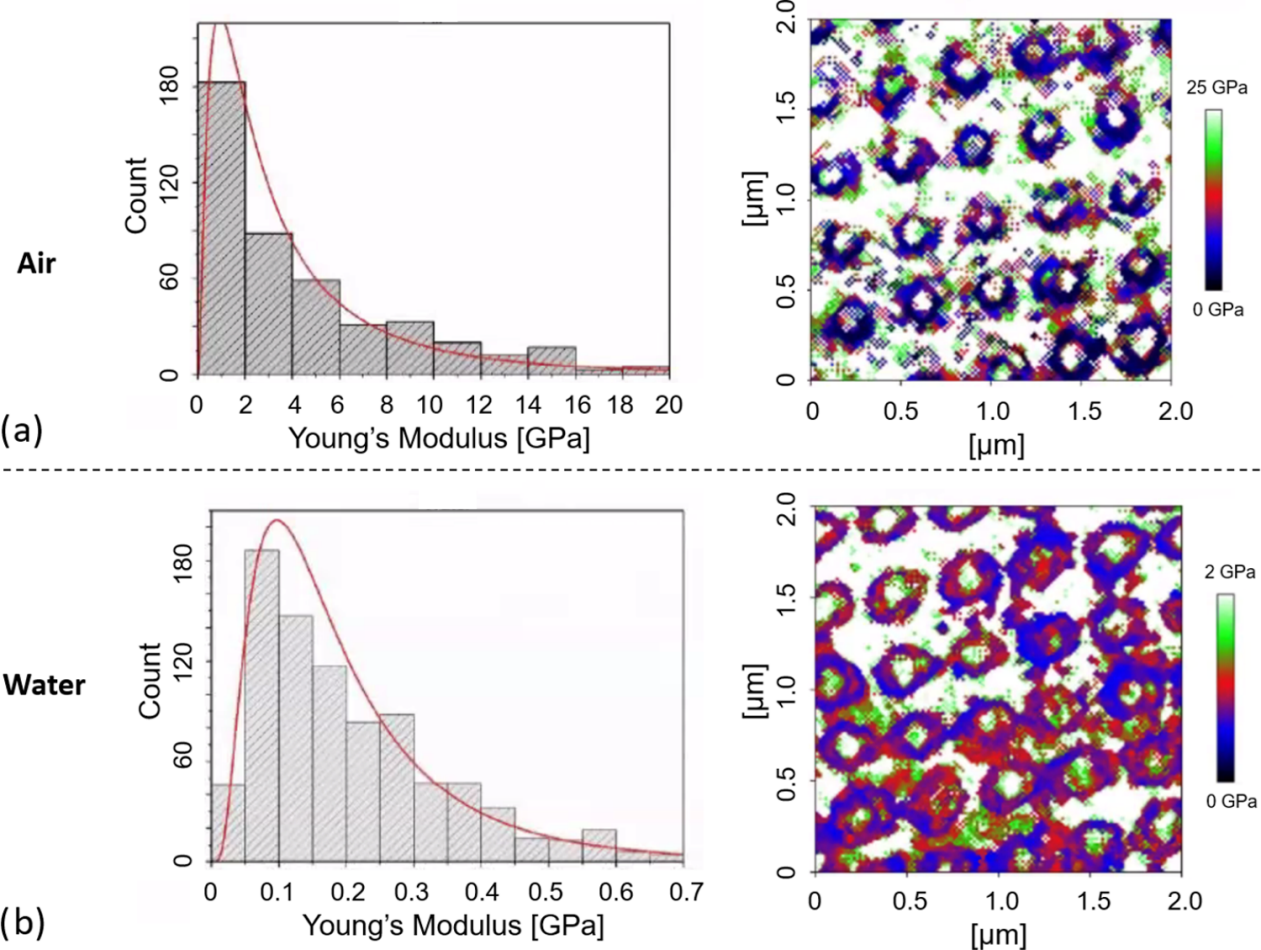
Comparison of Young’s moduli for the AuNP–polymer network hybrid system in (a) air and (b) water, with histograms on the left-hand side displaying Young’s moduli of 30 nanoparticles at random orientations and its corresponding logarithmic fitting.

In order to rule out that the polymer features appearing after the irradiation with the fs pulsed laser beam are associated with the effect of burning and to confirm that PE-MPC leads to a polymer network that forms a hydrogel after swelling with water, elastic properties were evaluated by mapping of Young’s modulus in air and in water (see Figure 4a,b, respectively).

Both micrographs reveal that around the hard metallic nanoparticles (with Young’s modulus >20 GPa), a belt-shaped region with lower stiffness occurs. The evaluation of these data using the elastic contact model according to Hertz with the Sneddon extension for the tip geometry was carried out. It is clearly visible that Young’s modulus of the dry polymer network around the AuNPs is around 10 times higher than that for the swollen network immersed in water forming a hydrogel (0–25 GPa for air and 0–2 GPa for water). The values for both Young’s moduli in air and water were derived from the histograms of the hydrogel’s Young’s moduli at random positions (around 30 AuNPs). The maximum of the fitting, representing the most probable value, was then identified as 1.5 ± 0.2 GPa in air and 0.10 ± 0.01 GPa in water. In addition, the degree of swelling of the polymer network was determined by comparing the size of the hydrogel nanostructure (distance between upper and lower) in either air or water (*N* = 50) from AFM images in contact or QI mode (data not shown). The size of the hybrid features in air was 320.3 ± 2.6 nm, while in water, it was slightly larger at 349.2 ± 2.2 nm, which can be attributed to swelling.

It is worth noticing that selective crosslinking occurs only on substrates with λ_LSPR_ of the AuNPs matching the wavelength of the NIR fs laser beam λ_L_. This was demonstrated by employing enlarged AuNPs (*D* ≈ 210 nm; Figure S4a) with the correspondingly red-shifted LSPR^57^ that become detuned from λ_L_. This leads to a lower field amplitude enhancement factor |*E*/*E*_0_| at λ_L_ (as predicted by simulations in Figure S2c) and, therefore, to a weaker plasmonic enhancement effect for triggering the MPC. In this case, a continuous layer of hydrogel is formed around the particles for conditions above the threshold (as seen in Figure S4b), while below it, no polymer is attached over the entire surface.

The second, pNIPAAm-based, polymer network was attached to AuNPs by an NIR fs beam that was polarized along one of the axes of the AuNP arrays. The home-built lithographer setup was used, and this optical system was designed for “point-by-point” writing of desired structures. For this system, a laser power *P*_L_ of 15 mW and a dwelling time *t*_d_ of 3 s were previously reported to provide the energy dose above the threshold for direct (nonplasmonically enhanced) MPC for the same poly(NIPAAm_94_-*co*-MAA_5_-*co*-BPQAAm_1_) terpolymer.^55^ For PE-MPC, the AuNP array with *D* = 165 ± 10 nm, a height of 50 nm, and a period Λ of 400 nm was exploited, with the LSPR tuned to the used λ_L_ (see Figure S5). Subsequently, the configuration of the scanning NIR femtosecond beam was changed to a decreased power of *P*_L_ = 10 mW and a shortened dwelling time of *t*_d_ = 1 s, which is below the threshold for the direct MPC recording but above the threshold for PE-MPC. Figure S6b presents the AFM height topography image of an area with AuNPs bearing the attached pNIPAAm-based network that was dried at room temperature after the rinsing step. Apparently, it does not reveal clear localization of the polymer network around the AuNPs, which we ascribed to its high swelling ratio (>10) as documented by our previous work on direct MPC.^55^ This effect was previously observed in the case of other pNIPAAm-based hydrogel nanostructures attached to a solid substrate and attributed to the strong surface tension of the water–air interface, which deforms the elastic polymer network and leads to a planarization of the soft crosslinked area as it reaches the hydrogel layer during water evaporation.^63^ However, when the same structure is allowed to swell in water at room temperature and subsequently heated to a higher temperature above the pNIPAAm VPTT before drying, the original crosslinked pattern can be retained (this is schematically shown in Figure S7a). To confirm this, we dried the sample at an elevated temperature of *T* = 80 °C and the results are shown in Figure S7b–d. Specifically, as can be seen from Figure S7c, the hydrogel structure dried at a temperature above its VPTT partially retained the shape, which resembles the plasmonic hotspots (similar to Figure 3c in the case of poly(DMAA_96_-*co*-AAHAQ_4_) copolymer). However, more pronounced smearing of the prepared hydrogel shape occurs for the poly(NIPAAm_94_-*co*-MAA_5_-*co*-BPQAAm_1_) hydrogel when compared to poly(DMAA_96_-*co*-AAHAQ_4_), which is attributed to the difference in their swelling ratio (swelling of >10 was estimated for the pNIIPAm-based hydrogel, while only 2.3 was determined for pDMAA-based gel from data in Figure S3).

### Polarization-Resolved LSPR Measurements

AFM observation did not clearly reveal the presence of locally attached polymer networks at the AuNP hotspot for the second polymer poly(NIPAAm_94_-*co*-MAA_5_-*co*-BPQAAm_1_) with a lower amount of 1% incorporated photocrosslinker, which we attributed to the softer characteristics of the formed hydrogel associated with a lower crosslink density and high swelling ratio. Therefore, we took advantage of the thermoresponsive properties of this pNIPAAm-based hydrogel and monitored the redistribution of surface mass density around the AuNPs when modulating the temperature above and below the VPTT of pNIPAAm. The LSPR wavelength λ_LSPR_ shifts with changes in the refractive index of the medium surrounding the AuNPs with a magnitude that is proportional to the surface mass density of the polymer materials. As the writing NIR femtosecond beam was made polarized for the PE-MPC, the prepared hydrogel nanostructures are expected to be attached only to the AuNP walls that are perpendicular to the incident electric intensity vector of the writing beam (see the near-field distribution in Figure 2d). In the collapsed state, when the temperature of the structure is above the pNIPAAm VPTT, the polymer network should be compacted at these AuNP sides, while below the VPTT in the swollen state, a delocalization and wrapping over the whole AuNPs is expected owing to its high swelling ratio.

**Figure 5:**
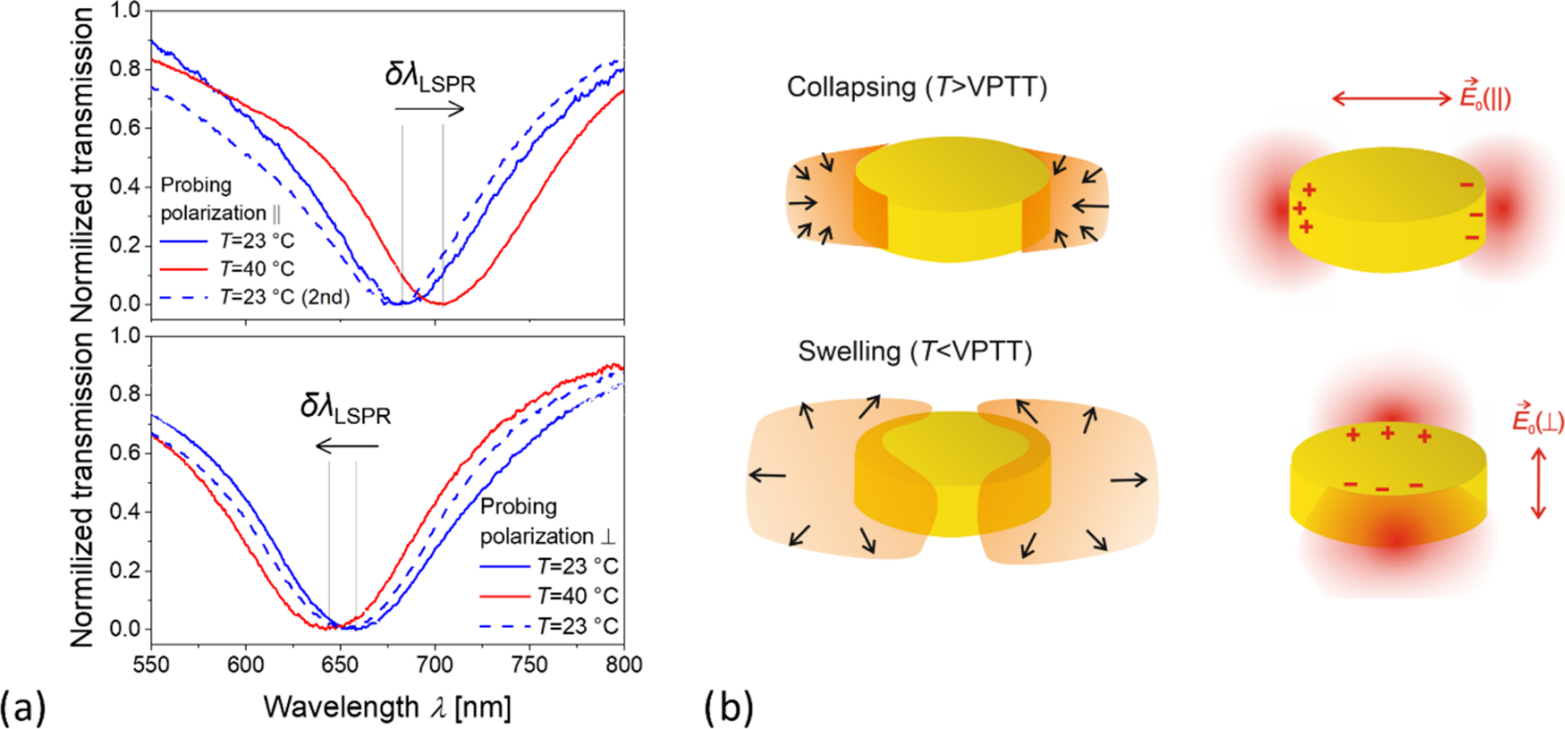
Polarization-resolved white-light transmission measurements of the plasmonically enhanced MPC-written poly(NIPAAm_94_-*co*-MAA_5_-*co*-BPQAAm_1_) sample: (a) normalized transmission spectra and (b) schematics of the hydrogel swelling and collapsing at the plasmonic hotspots.

In order to test the presence of the locally attached thermoresponsive pNIPAAm-based hydrogel to AuNPs, transmission LSPR spectra were measured with control over the polarization of the impinging optical beam at varying temperature *T*. As shown in Figure 5, the polarization of the probing optical beam was noted as “||-pol.” when it is made parallel to, and “⊥-pol.” when perpendicular to the polarization of the writing NIR fs laser beam. The substrate carrying AuNPs with the attached pNIPAAm-based nanostructures was mounted on a transparent temperature-controlled flow cell with water in contact with the surface.

First, measurements were done at room temperature (*T* = 23 °C), then the temperature was increased to *T* = 40 °C above the pNIPAAm VPTT, and finally, it was decreased again to room temperature (*T* = 23 °C, second). The corresponding measured transmission spectra in Figure 5a show that as the temperature increases above the pNIPAAm VPTT of 32 °C, the LSPR wavelength λ_LSPR_ for ||-pol. (top) red-shifts, while that for ⊥-pol. (bottom) blue-shifts. These shifts are of opposite sign and indicate that the collapse of pNIPAAm leads to asymmetrical redistribution of polymer networks around the AuNPs as an increase in the refractive index on the AuNP walls probed by LSPs with dipole moment parallel to the polarization (||) of the NIR fs laser beam occurs, while on the surface probed by the perpendicular orientation of the polarization (⊥), the refractive index decreases. This unusual behavior can be explained by assuming that the highly swollen pNIPAAm-based hydrogel nanostructure wraps around the whole AuNP in its swollen state. When increasing the temperature *T* above the LCST, the hydrogel compacts at the AuNP walls where it was attached by the plasmonically enhanced intensity of the NIR fs laser beam (Figure 5b). This effect is accompanied by an increased surface mass density and a higher refractive index of the hydrogel on walls perpendicular to the polarization (||) of the writing beam. On the surface of walls parallel to the fs NIR beam polarization (⊥) where the polymer network is not attached, it therefore retracts toward the plasmonic hotspots and is replaced by water with a lower refractive index of *n*_H_2_O_ = 1.33. Importantly, these transitions are reversible, and as the structure is cooled back to *T* = 23 °C, which is below the VPTT, the LSPR wavelengths shift to their original position. The magnitude of the LSPR shift for the || probing polarization is Δλ_LSPR_ = 22 nm (which is close to that measured for a pNIPAAm film that covers the whole area of similar AuNP arrays^64^), and it corresponds to the refractive index change of Δ*n*_h_ = 0.11 with a respective swelling ratio of about 7.5. The measured LSPR shift for the orthogonal polarization ⊥ is of Δλ_LSPR_ = −14 nm and corresponds to the refractive index change of Δ*n*_h_ = −0.07. This change would translate to a refractive index of the swollen hydrogel of *n*_h_ = 1.40, which corresponds to a lower swelling ratio and indicates that local gradients of the swelling process occurring in a quasi-three-dimensional manner play a significant role. Let us also note that the water surrounding the structure also changes its refractive index with temperature with a thermo-optical coefficient of d*n*_H_2_O_/d*T* = −1.2 × 10^–4^ RIU/K, but this effect can be neglected as it is associated with a much smaller decrease in the resonant wavelength of −0.4 nm for the temperature change of 17 K.

## Conclusions

Selective attachment of polymer networks forming a hydrogel at the plasmonic hotspot regions of metallic nanostructures is achieved. The obtained hybrid polymer–metallic nanostructure was prepared with nanoscale spatial precision by plasmon-enhanced multiphoton crosslinking of the presynthesized polymer chains conjugated with photoactive crosslinkers. With the use of optical configurations already established for a two-photon lithography relying on optically triggered polymerization, the reported alternative approach based on polymer crosslinking^55^ provides a facile means for local attachment of the functional hydrogel materials at the areas, where the electromagnetic field is strongly confined. This functionality is highly relevant in the fields of optical bioanalytical technologies and optical spectroscopy that take advantage of the plasmonically enhanced output optical signal. The prepared polymer network can be converted into a hydrogel serving as a three-dimensional large binding capacity matrix when postmodified with biorecognition elements.^65,66^ In plasmonic bioanalytical tools, such a binding matrix is designed to efficiently capture target analyte molecules from an analyzed liquid sample contacted with the sensor surface. In combination with the thermoresponsive properties, the optical readout sensitivity can be improved by using a hydrogel matrix that in the swollen state binds the target analyte inside its open network structure followed by its compacting at the plasmonic hotspot via a thermally induced network collapse performed prior to the optical readout. This approach provides dual amplification means, and it was already investigated with a thin pNIPAAm-based hydrogel binding matrix layer collapsing in the direction perpendicular to the surface in conjunction with the optical probing by surface plasmons propagating along the thin metallic film.^67^ When using the tighter confined and stronger amplified LSP field generated on metallic nanoparticles, the performance of such a sensor can simultaneously capitalize on stronger optical enhancement and on compacting the captured analyte species at narrower and sparser distributed plasmonic hotspots via a collapse in both perpendicular and parallel directions. The proposed approach may pave the way to such a new class of hybrid plasmonic structures tailored for the optical biosensor and spectroscopy applications with improved accuracy.^66^

## Data Availability

The data that support this study are openly available at 10.17605/OSF.IO/V52GB.

## Supporting Information Available

Additional experimental details and results of electromagnetic numerical simulations, AFM topography of control structures, and used thin film characteristics (PDF)

## Abbreviations

AAHAQ: acylamido-3-hydroxyanthraquinone
AFM: atomic force microscopy
AuNP: gold nanoparticle
BPdiS: 3,3′-disulfanediylbis(*N*-(4-benzoylbenzyl)propanamide)
BPQAAm: quaternary ammonium cation *N*-(4-benzoylphenyl)acrylamide
DMAA: dimethylacrylamide
DMSO: dimethyl sulfoxide
FDTD: finite-difference time-domain
LCST: local critical solution temperature
LSP: localized surface plasmon
LSPR: localized surface plasmon resonance
MAA: methacrylic acid
MPC: multiphoton crosslinking
NIPAAm: *N*-isopropylacrylamide
NIR: near-infrared
NP: nanoparticle
PE-MPC: plasmon-enhanced multiphoton crosslinking
QI: AFM quantitative imaging mode
VPTT: volume phase transition temperature
