# Supplementary material for: Plasmon-Enhanced Multiphoton Polymer Crosslinking for Selective Modification of Plasmonic Hotspots
Source: J Phys Chem C Nanomater Interfaces. 2024 Oct 22;128(43):18641–50. doi: 10.1021/acs.jpcc.4c05936 (PMC11533195; doi:10.1021/acs.jpcc.4c05936)
Supplement: Supplementary file 1 — jp4c05936_si_001.pdf [file jp4c05936_si_001.pdf]

Supporting information to:

Plasmon-Enhanced Multiphoton Polymer Crosslinking for Selective Modification of Plasmonic Hotspots

Yevhenii M. Morozov,[†*] Nestor Gisbert Quilis,[||] Stefan Fossati,[ↆ] Laura de Laporte,[Δ] Claudia Gusenbauer,[§] Andreas Weber,[▲] Jose Luis Toca-Herrera,[▲] Fiona Wiesner (née Diehl),[‡] Ulrich Jonas,[‡] and Jakub Dostalek[ↆ#*]

[†] Center for Health & Bioresources, AIT-Austrian Institute of Technology, Giefinggasse 4, Vienna, 1210, Austria

[||] Biosensor Technologies, AIT-Austrian Institute of Technology, Konrad-Lorenz-Strasse 24, Tulln an der Donau, 3430, Austria

[ↆ] FZU-Institute of Physics, Czech Academy of Sciences, Na Slovance 2, Prague, 182 21, Czech Republic

[Δ] DWI-Leibniz Institute for Interactive Materials, Forckenbeckstrasse 50, Aachen, 52056, Germany

[§] Institute of Wood Technology and Renewable Materials, University of Natural Resources and Life Sciences, Vienna, Konrad-Lorenz-Strasse 24, Tulln an der Donau, 3430, Austria

[▲] Institute of Biophysics, University of Natural Resources and Life Sciences, Vienna, Muthgasse 11/II, Vienna, 1190, Austria

[‡] Macromolecular Chemistry, Department of Chemistry and Biology, University of Siegen, Adolf Reichwein-Straße 2, Siegen, 57074, Germany

[#] LiST-Life Sciences Technology, Danube Private University, Viktor-Kaplan-Strasse 2, Wiener Neustadt, 2700, Austria

* Correspondence should be directed to: Yevhenii.Morozov@ait.ac.at; dostalek@fzu.cz

Characterization of the threshold for burning

The observation was carried out for pDMAA-based polymer network fabricated with the Nanoscribe system as can be seen in Figure S1.

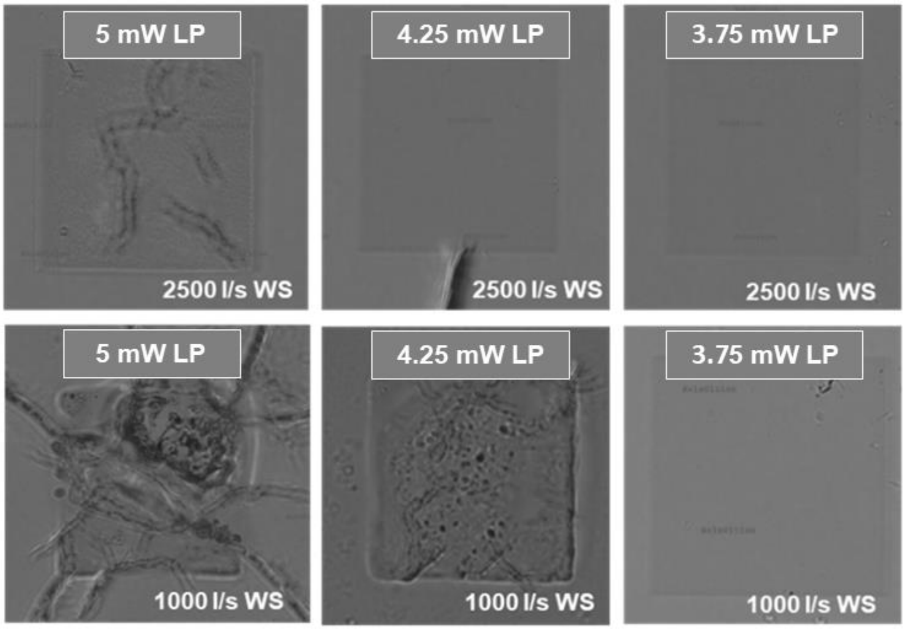

Figure S1. Optical microscope images of the inscribed square-shaped structures with varying laser power (LP) and writing speed (WS) of the poly($DMAA_{96}$-*co*-$AAHAQ_4$) polymer.

Near-field optical simulations

Figure S2 illustrates a trend in the near-field enhancement of electric field intensity in vicinity to arrays of AuNPs with increasing diameter *D*.

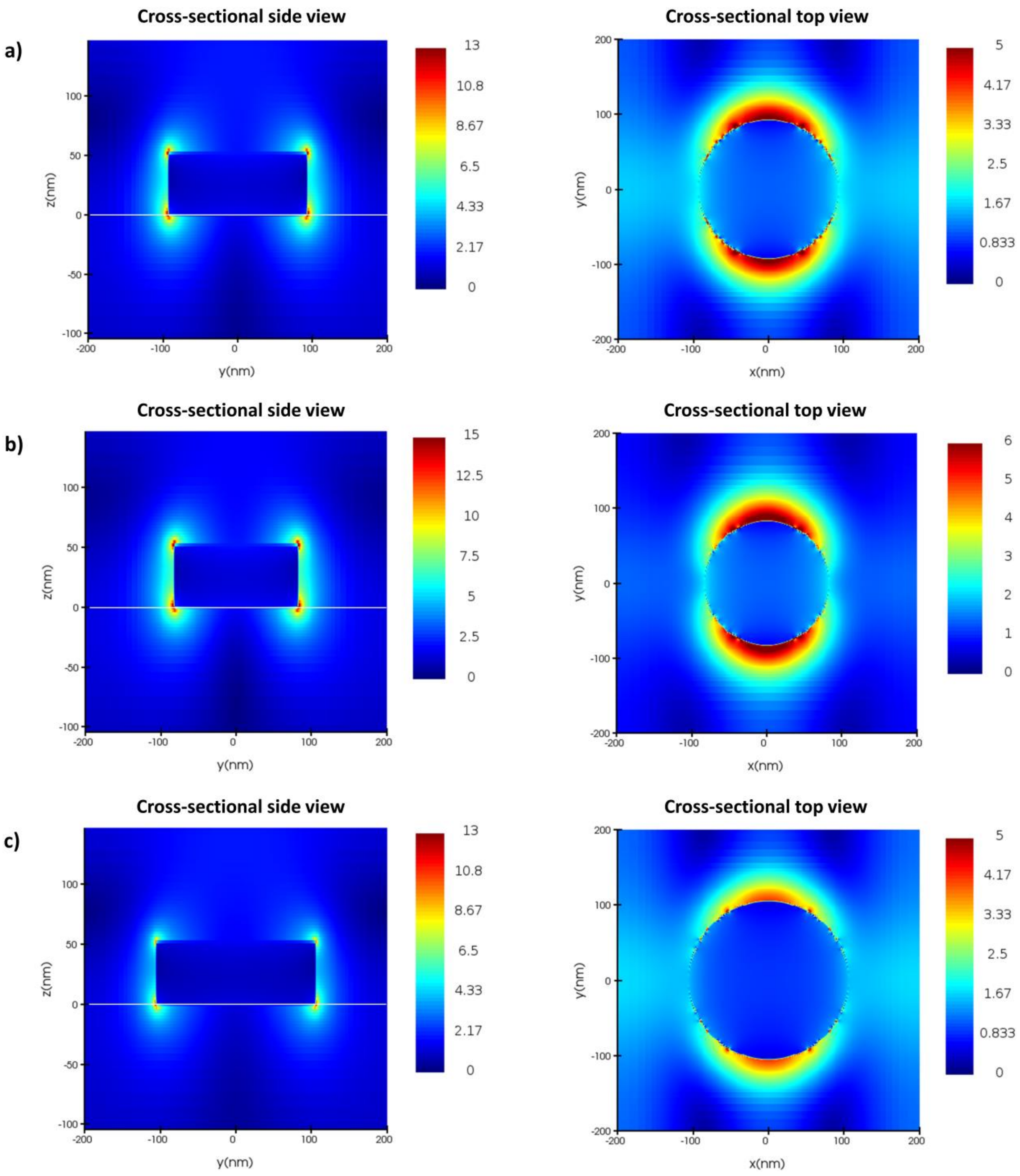

Figure S2. Lumerical FDTD simulation results for AuNP array having (a) D = 185 nm at λ = 780 nm; (b) D = 165 nm at λ = 785 nm; and (c) D = 210 nm at λ = 780 nm. The background refractive index was set to 1.45. All cross-sections were made through the NP centers. Electric field magnitude values are normalized to those of the incident (excitation) plane wave.

Determination of the 1D swelling ratio of the poly($DMAA_{96}$-*co*-$AAHAQ_4$) copolymer

Figure S3 demonstrates the angular reflectivity spectra measured for poly($DMAA_{96}$-*co*-$AAHAQ_4$) copolymer film with a home-built angular surface plasmon resonance setup.[1] The polymer films were prepared on the top of a flat 50 nm thick gold film that was modified with a self-assembled monolayer formed from benzophenone-disulfide. These spectra were fitted with a Fresnel reflectivity-based model. For a dry layer in contact with air (Figure S3a), a thickness of 154 nm was determined for the assumed refractive index of 1.45. Subsequently, the reflectivity spectrum was recorded for the same polymer film that was swollen in water (Figure S3b) and a thickness of 357 nm and a refractive index of 1.35 was determined under the assumption that the polymer surface mass density does not change. These results translate to a swelling ratio of 2.3 was determined between the dry and swollen layers in the perpendicular direction to the surface (1D swelling). The photographs of a suspended poly($DMAA_{96}$-*co*-$AAHAQ_4$) copolymer film in air and water are shown in Figure S3c.

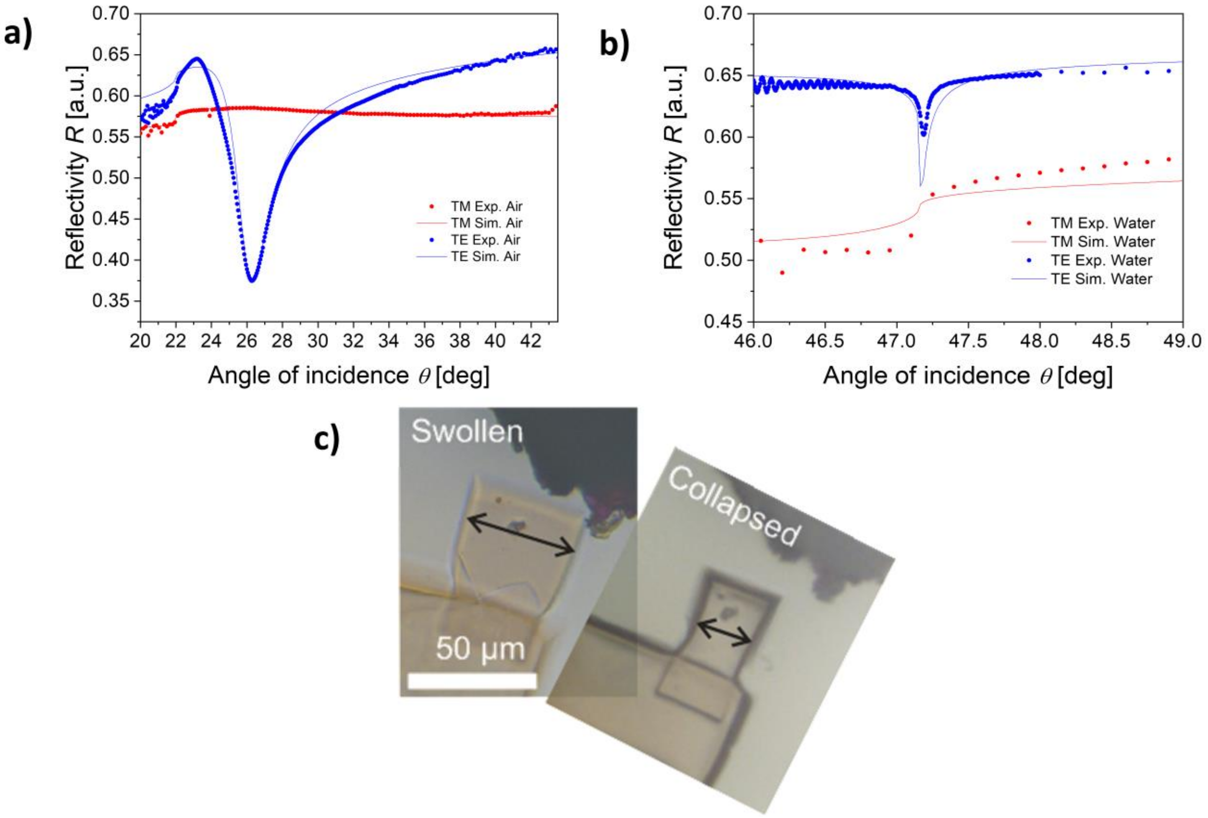

Figure S3. Angular reflectivity spectra measured for poly($DMAA_{96}$-*co*-$AAHAQ_4$) copolymer film in (a) dry and (b) swollen in water. (c) Photographs of the suspended poly($DMAA_{96}$-*co*-$AAHAQ_4$) copolymer film in air and water.

Control MPC experiment without matching λ_{LSP} with λ_L

MPC-prepared structures on the substrate, where the LSPR wavelength was not aligned with that of the NIR fs laser was characterized with AFM as presented in Figure S4.

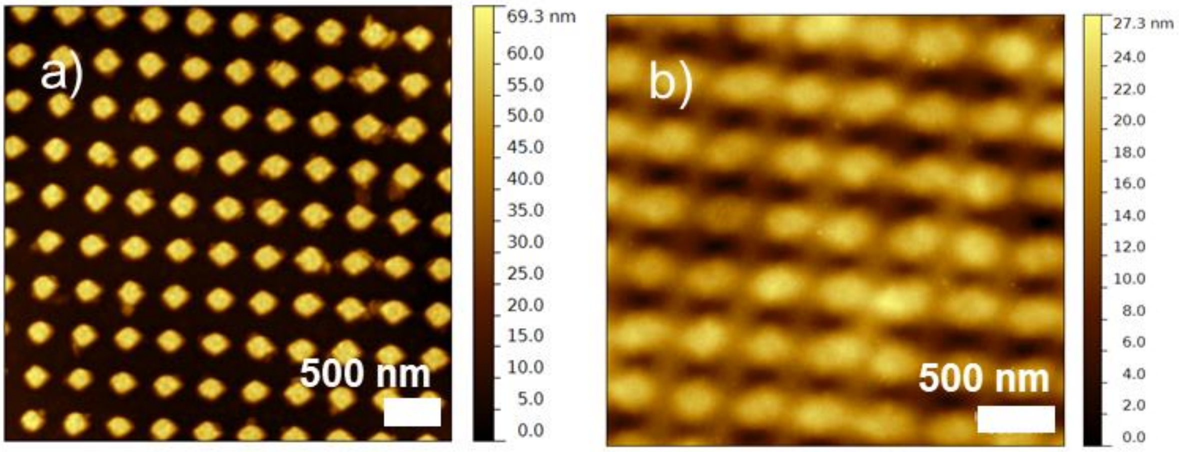

Figure S4. (a) AFM image of the bare AuNP array with AuNPs diameter of 210 nm; (b) AFM image of the poly(DMAA$_{96}$-*co*-AAHAQ$_4$) covered AuNP array irradiated with a laser power of 3.75 mW and a writing speed of 2,500 lines per second and rinsed with ethanol.

Experimental observation of LSPR shift due to the deposition of polymer layer

Topography of bare arrays of AuNPs were characterized by AFM (Figure S5a) and then the transmission LSPR spectra were measured before and after the pNIPAAm-based polymer layer deposition (Figure S5b)

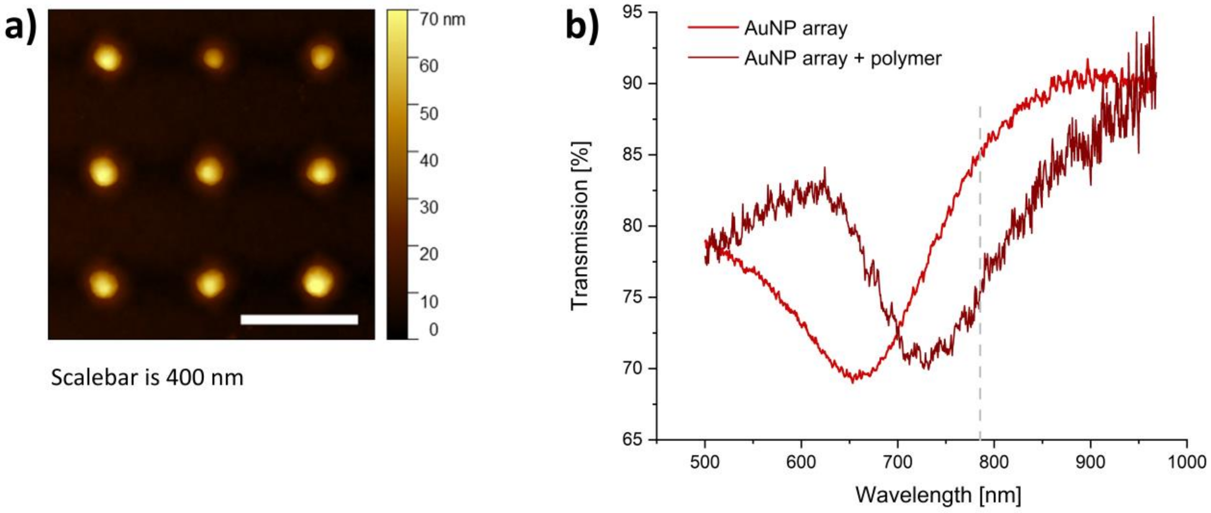

Figure S5. (a) AFM image of the bare AuNP array with AuNPs diameter of 165 ± 10 nm; (b) Corresponding white-light transmission spectra of bare AuNP array and AuNP array covered with a 100 nm layer of poly(NIPAAm$_{94}$-*co*-MAA$_5$-BPQAAm$_1$) terpolymer; dashed grey line indicates the fs laser wavelength λ_L.

Characterization of the PE-MPC with pNIPAAm-based polymer network.

Figure S6 illustrates the measurements with preparation of PE-MPC samples with pNIPAAm-based hydrogel where a signaling strip was written by using conditions above the threshold (so the polymer was crosslinked over the whole area in between AuNPs). Figure S7 provides details on AFM observation of height topography of the collapsed and dried pNIPAAm-based network locally attached to AuNPs depending on the drying temperature.

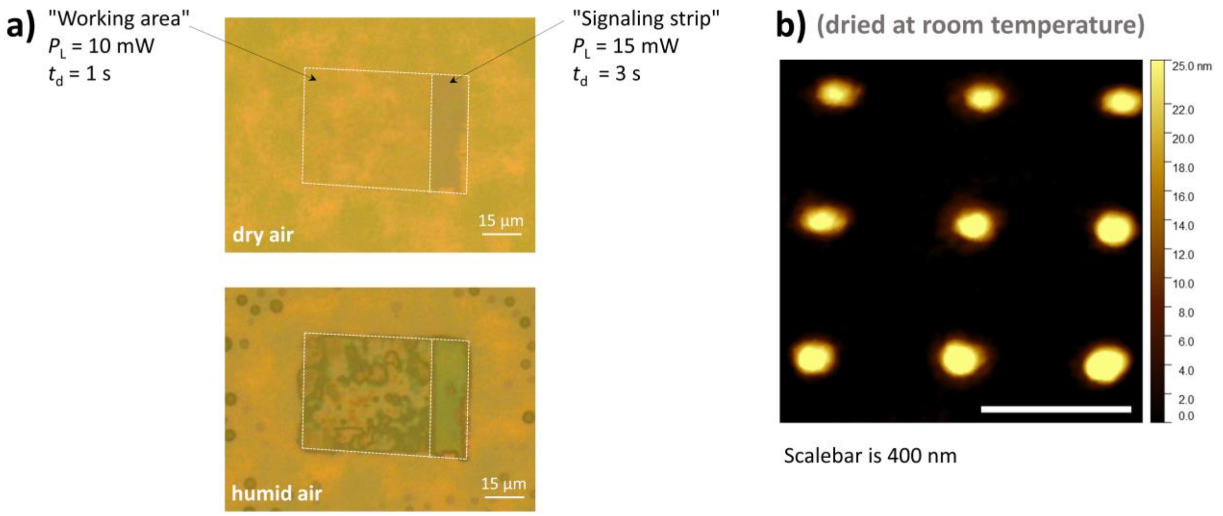

Figure S6. Plasmonically enhanced MPC experiment with the thermoresponsive poly(NIPAAm$_{94}$-*co*-MAA$_5$-BPQAAm$_1$) terpolymer. (a) Bright-field microscopy image of the written "patch" in the polymer layer in dry and humid air; (b) AFM image of the "working area" of the sample after the recording dried at room temperature.

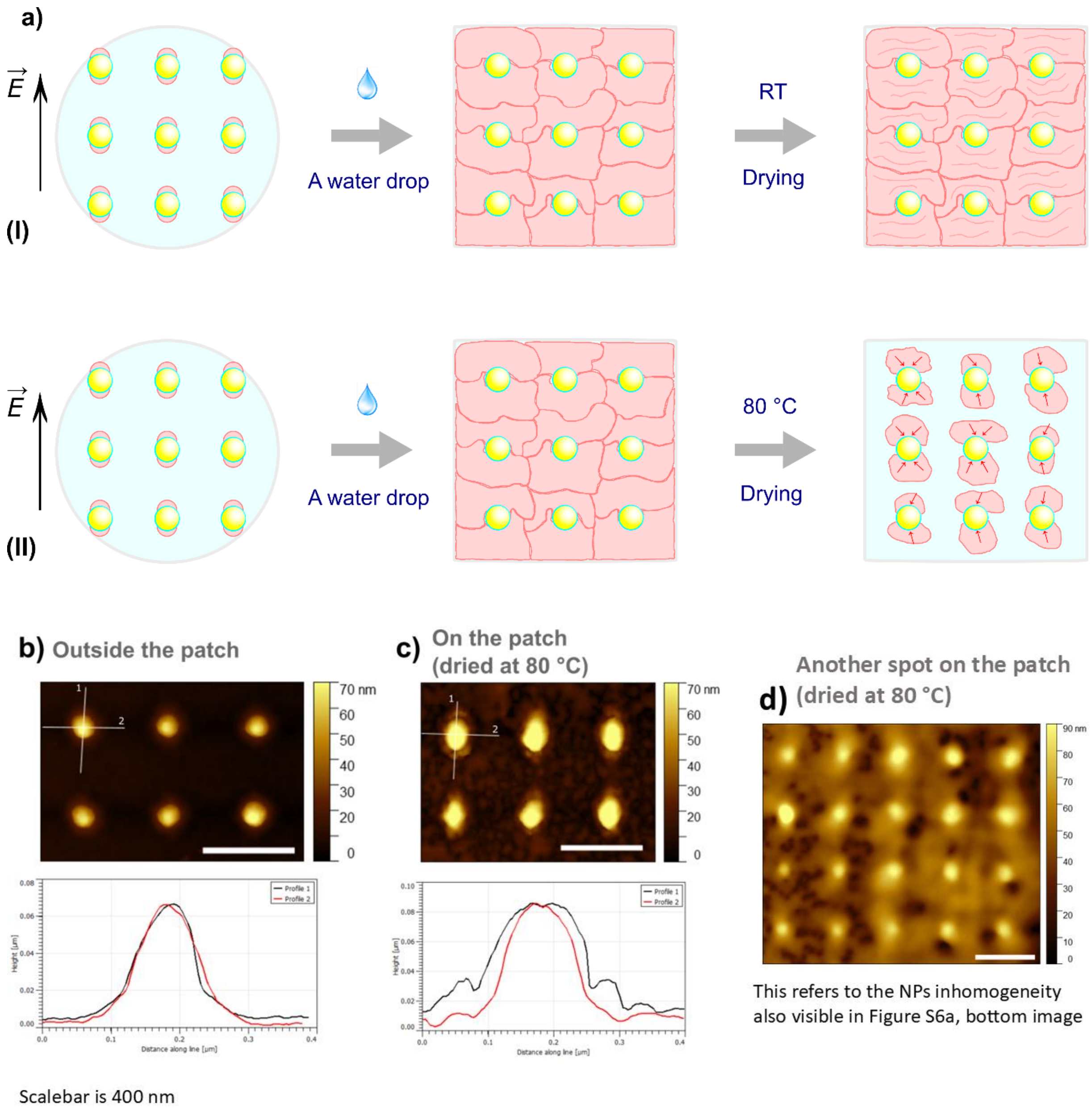

Figure S7. (a) A schematic showing the mechanism of the hydrogel shrinkage during drying at different temperatures: (I) at room temperature – the hydrogel shrinks slightly only in the vertical (perpendicular to the sample surface) direction; (II) at the elevated temperature of 80 °C – the hydrogel shrinks significantly also in the horizontal (in the sample plane) direction to its original shape; the black arrow indicates the polarization of the laser beam during plasmonically enhanced MPC. (b) AFM image of the sample outside the patch. (c)-(d) AFM images of the "working area" at different spots of the sample dried at the elevated 80 °C temperature.

References

(1) Morozov, Y. M.; Diehl, F.; Grün, J. J.; Pertiller, M.; Fossati, S.; Schmidt, K.; Quilis, N. G.; Gusenbauer, C.; Zbiral, B.; Toca-Herrera, J. L.; Klees, S.; Thiagarajan, C. R. V.; Jonas, U.; Dostalek, J. Microstructuring of thermoresponsive biofunctional hydrogels by multiphoton photocrosslinking. *Adv. Func. Mat.* **2024**, *34* (26), 2315578.
